# A WS_2_ Case Theoretical Study: Hydrogen Storage Performance Improved by Phase Altering

**DOI:** 10.1186/s11671-020-03337-6

**Published:** 2020-05-07

**Authors:** Jing Zhou, Jiamu Cao, Jianing Shi, Yufeng Zhang, Junyu Chen, Weiqi Wang, Xiaowei Liu

**Affiliations:** 1 MEMS Center, Harbin Institute of Technology, Harbin, 150001 China; 2 Key Laboratory of Micro-systems and Micro-Structures Manufacturing, Ministry of Education, Harbin, 150001 China

**Keywords:** First-principles, Hydrogen adsorption, Monolayer WS_2_, Phases, Hydrogen storage

## Abstract

Hydrogen is a clean energy with high efficiency, while the storage and transport problems still prevent its extensive use. Because of the large specific surface area and unique electronic structure, two-dimensional materials have great potential in hydrogen storage. Particularly, monolayer 2H-WS_2_ has been proven to be suitable for hydrogen storage. But there are few studies concerning the other two phases of WS_2_ (1T, 1T′) in hydrogen storage. Here, we carried out first-principle calculations to investigate the hydrogen adsorption behaviors of all the three phases of WS_2_. Multiple hydrogen adsorption studies also evaluate the hydrogen storage abilities of these materials. Comprehensive analysis results show that the 1T′-WS_2_ has better hydrogen storage performance than the 2H-WS_2_, which means phase engineering could be an effective way to improve hydrogen storage performance. This paper provides a reference for the further study of hydrogen storage in two-dimensional materials.

## Introduction

Conventional hydrogen storing carries considerable risk due to its low ignition, wide range flammability, and embrittlement on steel [1, 2]. Although metal hydrides, such as CaH_2_, can store large amounts of hydrogen, they are not only flammable when wet but also expensive and difficult to reuse. Therefore, the search for a safe, economical, and effective hydrogen storage material has become a widespread concern [3]. Due to their large specific surface area and unique electronic properties, two-dimensional (2D) materials have been widely used in many fields such as photocatalytic water splitting, hydrogen evolution reaction, transistors, electroluminescent devices, gas storage, and gas adsorption [4–9]. For example, hydrogen adsorbing on graphene entails the rehybridization of carbon valence orbitals by transforming C–C *π* bond to C–H *σ* bond, which could induce the bandgap and magnetic moment around the Fermi level so that hydrogenation of graphene offers an exciting possibility to directly write electronic circuits at the atomic scale with predesigned patterns [10]. The success of graphene-based materials has also motivated researches towards other 2D materials applied in gas adsorption or storage [11–14]. More importantly, monolayer transition metal disulfide (TMD) materials especially have shown excellent performance in hydrogen storage [15].

The capacity for hydrogen storage could be evaluated by the adsorption strength of the gas molecules over the surface of the material [16]. Adsorption strength should not be too strong or too weak because the target gas molecules are difficult to separate from the material under strong adsorbing force or unsteadily adsorbed under weak adsorbing force [17]. The average binding energies per hydrogen molecule of suitable hydrogen storage materials is from − 0.2 to − 0.6 eV at room temperature (about 25 °C) [12]. However, original materials such as graphene or TMDs have a deficiency that their binding force to hydrogen molecules is too weak [18, 19]. Surface functionalization methods were usually taken to improve their hydrogen adsorbing properties. By doping or decorating process, the surface characteristics of 2D materials can be changed to fit the range of moderate hydrogen adsorption energy, and the hydrogen storage performance can be further improved [20]. However, it is difficult to maintain the stability of decoration systems [21, 22]. And it is challenging to dope or decorate accurately [23]. Such methods are theoretically feasible, but far from applications.

As typical TMDs, MoS_2_ and WS_2_ have proved their excellent application potential in the field of hydrogen storage [24, 25]. Because of its superior catalytic performance and unique electrical properties, MoS_2_ is widely concerned in many areas [26], and WS_2_ is often overlooked. Compared with single-layer MoS_2_, WS_2_ has better thermal stability [27, 28] and greater binding energy with hydrogen molecules under compression strain [29]. It is known that WS_2_ also has two other phases (1T/1T′), which have distinct symmetries and different electronic properties. Previous studies have shown that they can be prepared in simple methods [30, 31]. Most methods were based on phase transition from 2H phase WS_2_ and combined with stabilization ways. Many studies have shown successful preparation of high-percentage and stable 1T/1T′-WS_2_ (Table S1). Recently, the metallic 1T-WS_2_ and its ramification 1T′-WS_2_ have demonstrated great potential in hydrogen evolution reaction (HER) applications [23, 32]. The research results presented that their surface has moderate adsorption strength to the reaction intermediate H*. That paves the way for their other applications that related to the adsorption of hydrogen such as hydrogen storage. However, there are few studies concerning hydrogen storage properties of these two phases of WS_2_. The effects of phase difference on hydrogen storage have always been ignored.

In this work, we investigated all the three phases of WS_2_ to compare their suitability to be a hydrogen storage material. We performed a systematic theoretical study of the structures and analyzed the adsorption energy and adsorption configuration of gas molecules. To simulate real working conditions, the adsorption of numerous hydrogen molecules was studied. With the calculation results in this work, we found that 1T′-WS_2_ is the best candidate among these three phases of WS_2_ as a hydrogen storage material. Altering the phase of WS_2_ gives an improvement in hydrogen storage. Thus, it can provide a reference for the research of hydrogen storage by two-dimensional materials in the view of phase.

## Computational Details

The first-principles were used based on the density functional theory (DFT). All calculation in this work was conducted in Dmol3 [33]. The local density approximation (LDA) is used to handle exchange and correlation potentials with the PWC function. The single productive potential was used to replace kernel (DFT semi-core pseudopots) to reduce the computation cost. Higher accuracy was achieved by choosing a dual numerical orbital basis set and orbital polarization function (DNP). Then, a convergence test was given. After the test, the Monkhorst-Pack *k*-points was set to 4 × 4 × 1, then make a vacuum layer of 20 Å to prevent interlayer interactions. The energy convergence precision was set to 1 × 10^−5^ Hartree (1 Hartree = 27.212 eV), the max displacement was 0.005 Å, and the atomic forces were not over 0.002 Hartree/Å. All the later calculations follow these properties.

For these three phases of WS_2_ (1T/1T′/2H), the calculation models were supercells of 4 × 4 monolayer WS_2_. The 1T-WS_2_ and 2H-WS_2_ structures were first constructed by ourselves. After the construction is completed, geometric optimization is carried out, including unit optimization. And the 1T′-WS_2_ was built based on the existed 1T′-MoS_2_. While the 1T′-MoS_2_ was built based on a 2 × 2 1T model, a single hydrogen atom was set key joint to an S atom of the 1T MoS_2_. Then, the system was given another geometry optimization. After optimization, the hydrogen atoms were removed and optimized again to obtain the regular 1T′-MoS_2_ structure. After that, all the Mo atoms were replaced by W atoms in a 2 × 2 model, then went through a geometry optimization, including cell optimization again.

With the optimized 2 × 2 WS_2_ model, a supercell of 4 × 4 monolayer WS_2_ was constructed. As shown in Figure S1, the models of all these three phases of WS_2_ contain 32 S atoms and 16 W atoms in a cell. Because 16 of the 25 W atoms presented in the 1T phase model are at the sites of the edge or corner, the valid quantity of W atoms in the cell is still 16. The bonds between each W atoms in 1T or 2H model are equal while those in 1T′-WS_2_ are not equal. With the W–W bond in 1T′-WS_2_, the W atoms’ arrangement looks like a zigzag-chain.

**Fig. 1:**
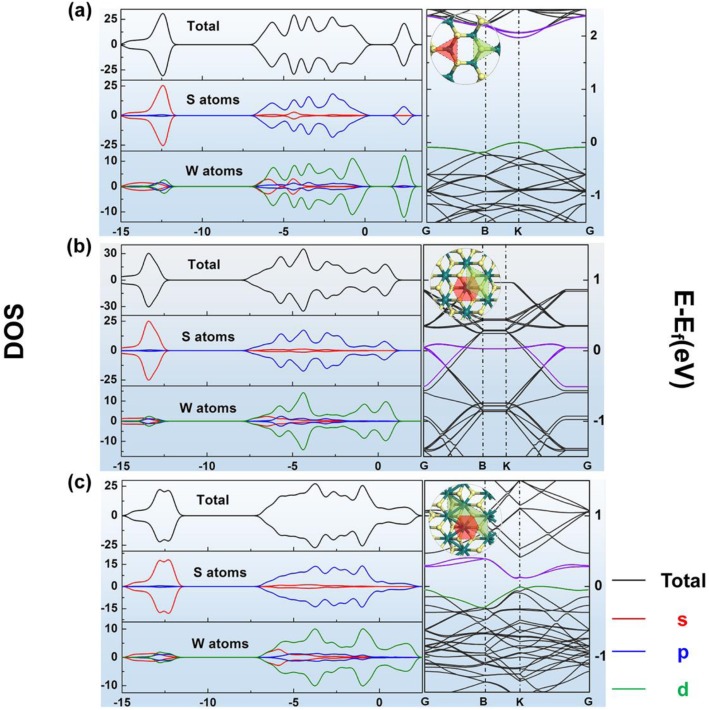
Geometry structure, DOS, and band structure results of **a** 2H-WS_2_, **b** 1T-WS_2_, and **c** 1T′-WS_2_; yellow balls represent S, and glaucous balls represent W

Consequently, the 1T′ phase is also called the zigzag-chain phase in some studies. We can find repeat units in the three structures that have characters in common. As shown in the illustrations of Fig. 1, the green boxes represent the repeating units with only W atoms on the edge, while the red ones are those outlined by S atoms. Owning to the symmetry difference, the size of the green box in the 1T′ model is nearly twice as big as the one in the 1T model. The red box in 1T or 1T′ models is a hexagon, but in the 2H model, it is a triangle. There are also similar repeating units in 1T and 1T′-WS_2_ structures, such as the blue rectangular area in Figure S1. Besides, the axisymmetric elements shown in the red boxes in the 1T and 1T′ models can also be found in Figure S1 and that could also represent the symmetry of 1T and 1T′-WS_2_ structure.

A single hydrogen molecule was placed on the *c*-axis above the WS_2_ plane to establish a hydrogen adsorption model, and several adsorption sites with high geometric symmetry were selected. For the case of 1T-WS_2_ shown in Figure S2 (b) and (e), there were five sites: just over the upper layer S atom, just over the lower layer S atom, just over the W atom, above the bond of W atom, and the upper layer S atom, above the bond of W atom and lower layer S atom. And for 1T′-WS_2_, these six situations were shown in Figure S2 (c) and (f). For 2H-WS_2_ shown in Figure S2 (a) and (d), there were four situations: just above the S atom site, just above the W atom site, above the middle of the W atom and S atom site, and right above the center of the hexagon structure. These sites were chosen since they are highly symmetric sites of these materials. After given geometry optimizations and comparison of adsorption energy, stable adsorption sites could be found. And we distinguished the posture of the hydrogen molecule adsorbed on the 1T′-WS_2_ because of its relatively lower structural symmetry. The hydrogen molecules were set either horizontally or vertically (as shown in Figure S3), which doubled the situation. After the geometry optimization, all the adsorption energy is presented in Table S2. The most stable adsorption sites were chosen according to the adsorption energy results. For the hydrogen adsorption process, the adsorption energy is calculated by the following function: *E*_ad_ = *E*_tot_ − *E*_mat_ − *E*_hyd_, where *E*_tot_ is the total energy of each of these three phases of WS_2_ with the hydrogen molecules adsorbed, *E*_mat_ (energy of the material) represents the total energy of pristine WS_2_, and *E*_hyd_ represents the total energy of an isolated hydrogen molecule. According to this relation, a higher absolute value of *E*_ad_ leads to more stability of the adsorption system. The acting force between the materials and target gas molecules also can be reflected by the absolute value of *E*_ad_. A repulsive force is represented by a positive value of *E*_ad_, while a negative value reflects an attractive force. Although the exact adsorption energy could not be gotten through this method [34], it can reflect the form and strength of the interaction between hydrogen and adsorbing material. As introduced above, the ideal adsorption energy for hydrogen storage applications for each hydrogen molecule is − 0.2 to − 0.6 eV/H_2_ at room temperature [35].

## Results and Discussion

**Fig. 2:**
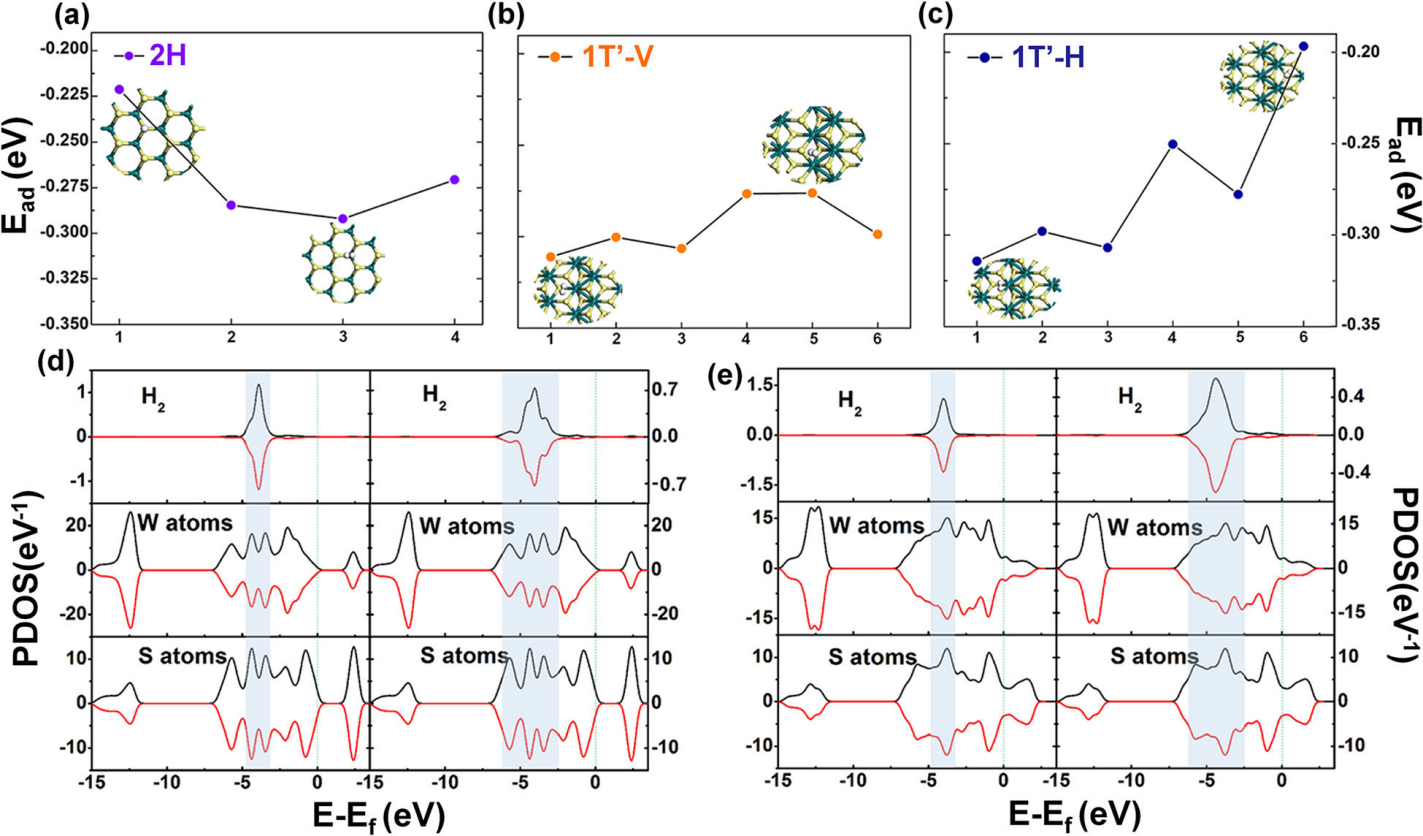
Adsorption energy results of H_2_ adsorption system for **a** single H_2_ on 2H-WS2, **b** and **c** single H_2_ on 1T’-WS_2_; PDOS results of the situation with thelowest (left) or the highest (right) E_ad_ in **d** 2H models and that in **e** 1T’ model

For all these materials’ models, the structures with the lowest energy could be found after the geometry optimization. The lengths of all the W–S bonds in monolayer 1T-WS_2_ and 2H-WS_2_ are 2.428 Å and 2.402 Å, respectively. But those in the 1T′-WS_2_ are unequal, which have lengths of around 2.453 Å, 2.410 Å, and 2.490 Å. It can also be found that W–W bonds in the optimized 1T′ model have a length of about 2.784 Å. Band structures of all these three phases of optimized pristine WS_2_ are shown in Fig. 1. For the metallic 1T phase, there is no bandgap. And for 1T′ phase, it has a semi-metallic band structure. While in the 2H phase, the band structure agrees with the characteristic of a semiconductor. The partial density of state (PDOS) of these three models is also shown in Fig. 1. It can be seen from the PDOS results that the shape of S-p and W-d orbits are most similar to that of the total DOS in all these three figures, indicating S-p and W-d orbits contributed to the total DOS, mostly for all these three phases of WS_2_. The tendency of the DOS results of 1T′-WS_2_ is accordant to the band structure and agrees with the previous study [32]. The different positions of absorbed hydrogen molecules were compared to find the most stable ones in all these three models. Positions were chosen according to the *E*_ad_ and Hirschfeld charge results of the listed situations about the absorbed single hydrogen molecules in the structures of these three phases (the *E*_ad_ and Hirschfeld charge results are shown in Fig. 2a–c and Table S2). For 1T WS_2_, it is site 3, and for 1T′-WS_2_, it is site 1 (as shown in Fig. 2b, c), while for 2H-WS_2_, it is site 3 (all shown in Fig. 2a and Table S2-S3). Based on these results, firstly, 1T phase WS_2_ is not suitable for hydrogen adsorption because the *E*_ad_ for hydrogen on 1T WS_2_ is far significant than 0.6 eV (Table S2). That means it will be too hard to release the adsorbed hydrogen molecules from the 1T WS_2_ surface. According to this result, the following studies should not concern about this phase. The *E*_ad_ results of 1T′ phase and 2H phase are around − 0.27 eV, both are in the applicable adsorption energy range for hydrogen storage applications.

To further compare these two phases, the PDOS analysis was carried out, which is shown in Fig. 2d, e. The left parts show the lowest PDOS adsorption energies of the two phases, while the right parts are the two of the highest ones. There are small differences in both situations of the lowest or the highest energy. In the left part of Fig. 2d, e (which corresponding to the lowest adsorption energy), the main peaks are both at − 3 to − 5 eV. While for the right part (which represents the highest adsorption energy), it appeared between − 2.5 and − 6 eV. This appearance means there is a larger superposition between the PDOS of the hydrogen molecule and WS_2_, which indicates a stronger interaction between them. These results agree with the adsorption energy results well. However, the PDOS results for single-hydrogen molecule situations still could not well reflect the difference in hydrogen adsorption property between these two kinds of materials.

Therefore, we have done the study about different numbers of hydrogen molecules adsorbed on the surface of both 1T′ and 2H-WS_2_. As Figure S4 shows, we set different numbers of hydrogen molecules (16, 32, 48, and 64) on the surface of both 1T′ and 2H-WS_2_. For 1T′-WS_2_, when the number of hydrogen molecules is under 16, each of the hydrogen molecules is set in the most stable position (site 1v). Considering the influence of the potential interaction between multiple H_2_ molecules, we further discussed the arrangement of H_2_ when 2 or 3 H_2_ molecules adsorbed 1T′-WS_2_. For two hydrogen molecules, we considered three situations: neighboring sites (2H_2_-1), on separated sites of the same side (2H_2_-2), and on the closest sites of different sides (2H_2_-3). For three hydrogen molecules, there were five cases: three neighboring sites of the same side (3H_2_-1); two neighboring and one separated, all on the same side (3H_2_-2); three separated on the same side (3H_2_-3); two neighbors on the same side and one on the other side (3H_2_-4); and two separated on the same side and one on the other side (3H_2_-5). The calculated adsorption for each case was compared (Table S4). The results show that setting hydrogen molecules on neighboring sites of 1T′-WS_2_ would make the total adsorption energy larger than separated cases. That means irregular adsorption energy change will be brought if H_2_ molecules were set randomly even on the same adsorption site. However, there was no evident influence when hydrogen molecules were set on the closest sites of different sides of 1T′-WS_2_. Based on these results, hydrogen molecules are set according to the following principles: when H_2_ molecules are below 8, hydrogen molecules are set on nonadjacent adsorption sites on either side of 1T′-WS_2_; when the number is 8 to 16, neighboring sites could not be avoided. Adjacent adsorption sites are still avoided as much as possible. When hydrogen molecules are between 17 and 32, 16 of them are set at the most stable position (site 1v), and the rest are set vertically above the W atoms (site 3v). When hydrogen molecules are more than 32, the distance between these hydrogen molecules will be given priority to avoid forming the hydrogen molecular groups, which is shown in Figure S6. And then, the horizontal or vertical placement will depend on the adsorption energy results of single hydrogen. Therefore, when the H_2_ is between 33 and 48, the first 16 molecules are in site 1v, the second 16 molecules are in site 3v, and the rest are in site 4h. When the number is above 48, the first 16 molecules are in site 1v, the second 16 molecules are in site 3v, the third 16 molecules are in site 4h, and the rest are in site 2h. We try to arrange hydrogen molecules evenly on both sides of this structure and ensure that the distance between each hydrogen molecule is far enough. In the condition of the 2H phase, similar to the cases of 1T′-WS_2_, when hydrogen molecules are below 32, each one is set at the most stable position discussed above (site 3). To avert the effects of inconsistency caused by the interaction between hydrogen molecules, hydrogen molecules were set on nonadjacent sites when the amount is less than 16. But we should try to avoid neighboring sites when the amount is between 17 and 32. When the number is between 33 and 64, the rest is placed in the center of the hexagon (site 4). We also try to distribute all the molecules following the principle mentioned above. On the other hand, we also consider the stability of the adsorption system with a high concentration of H_2_ molecules. When a gas molecule is more than 16, the stability of the whole system has also been explored by molecular dynamics simulations, which is presented in Figure S7. After 500 steps of molecule dynamics simulation, there is no geometry buckling emerging, and the total energy also remains almost constant so that the whole system has great stability.

**Fig. 3:**
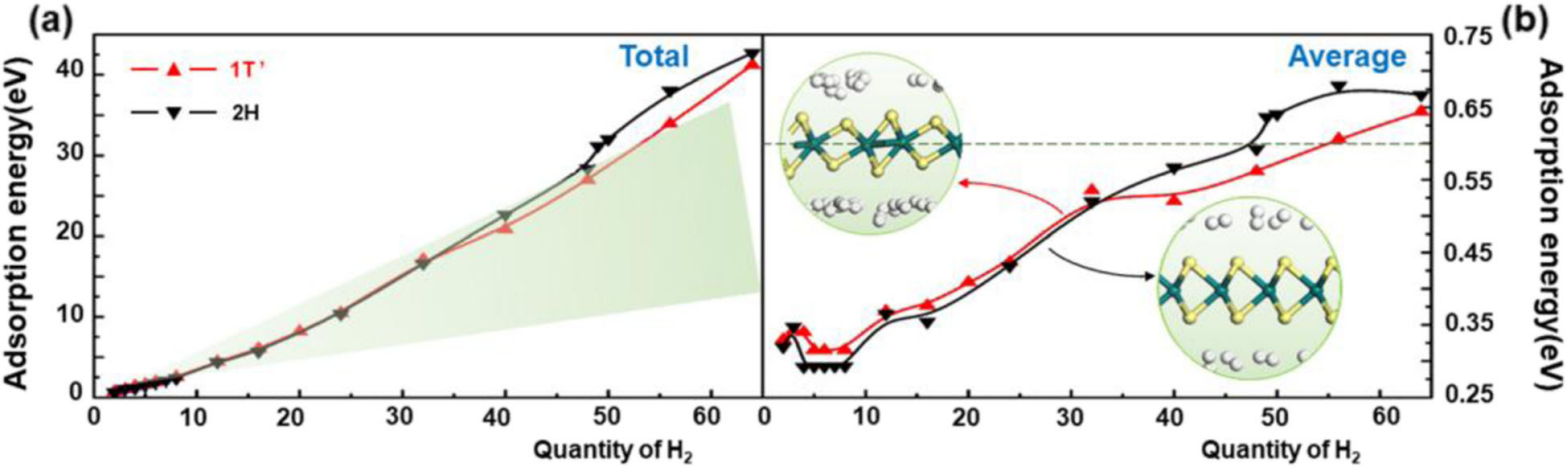
Graphs of **a** total adsorption energy and **b** average adsorption energy as a function of the number of hydrogen molecules absorbed on the 1T’- and 2H-WS_2_

The adsorption energy to the hydrogen molecules was calculated after given a geometry optimization. As Fig. 3 shows, no matter which phase of WS_2_, the total adsorption energy increases nearly linearly when the number of hydrogen molecules increases. That means when the number of hydrogen molecules increases, the interacting force between the material and the adsorbed molecules does not change much. The green area in Fig. 3a presents the moderate hydrogen adsorption energy area. It can be found that 2H-WS_2_ comes out of this area earlier than 1T′ phase. That means when the quantity of adsorbed H_2_ molecules become redundant, more hydrogen molecules will be hard to be released from 2H-WS_2_ than from 1T′-WS_2_, which intends a smaller hydrogen capacity. Then also, as Fig. 3 shows, the number of hydrogen molecules for the average adsorption energies of the adsorbed hydrogen molecules to be in the range of − 0.2 to − 0.6 eV is below 48 or 55 at the situation of 2H or 1T′ phase, respectively. That means the theoretical reasonable adsorption quantity for hydrogen on 2H-WS_2_ can be up to 2.4 wt%, while that on the 1T′ phase, it can be up to 2.7 wt%. That reveals changing the phase could enhance the hydrogen storage performance of WS_2_ effectively. The average adsorption energy of the two kinds of WS_2_ decreases and then increases when it is no more than 8. It is easy to understand that when the material adsorbs more gas molecules, the average interacting force between the gas molecules and the material will become weaker. However, when the number of hydrogen molecules is greater than 8, the reason for the increasing average adsorption energy is still unknown.

**Fig. 4:**
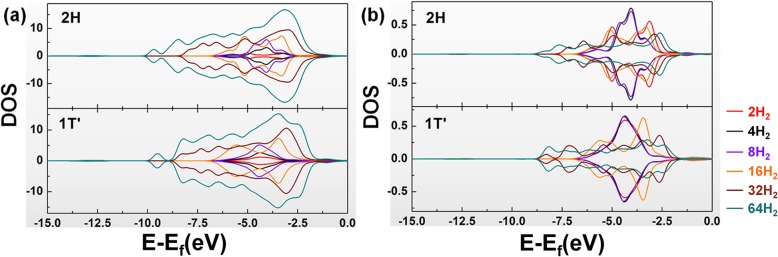
PDOS results of multiple H_2_ adsorption systems for _a_ all and _b_ single hydrogen molecules on 2H-WS_2_ and 1T’-WS_2_

Do the PDOS study again, as shown in Fig. 4. It can be found that as the number of hydrogen molecules increases, the total PDOS of the adsorbed hydrogen molecules becomes dispersed in both phases of WS_2_ (especially when the number of hydrogen molecules is more than 16). And the PDOS scope of single hydrogen molecules adsorbed in these systems also becomes more extensive. But the PDOS for the W atoms and S atoms remains unchanged, which represents the stability of these two materials when hydrogen molecules were adsorbed. The results also show that as the number of hydrogen molecules increases, the PDOS overlap area between the hydrogen molecules and the two WS_2_ molecules increases.

The interaction between hydrogen molecules and WS_2_ becomes stronger. That reveals the reason for the increase in the average adsorption energy when the number of adsorbed hydrogen molecules becomes more.

**Fig. 5:**
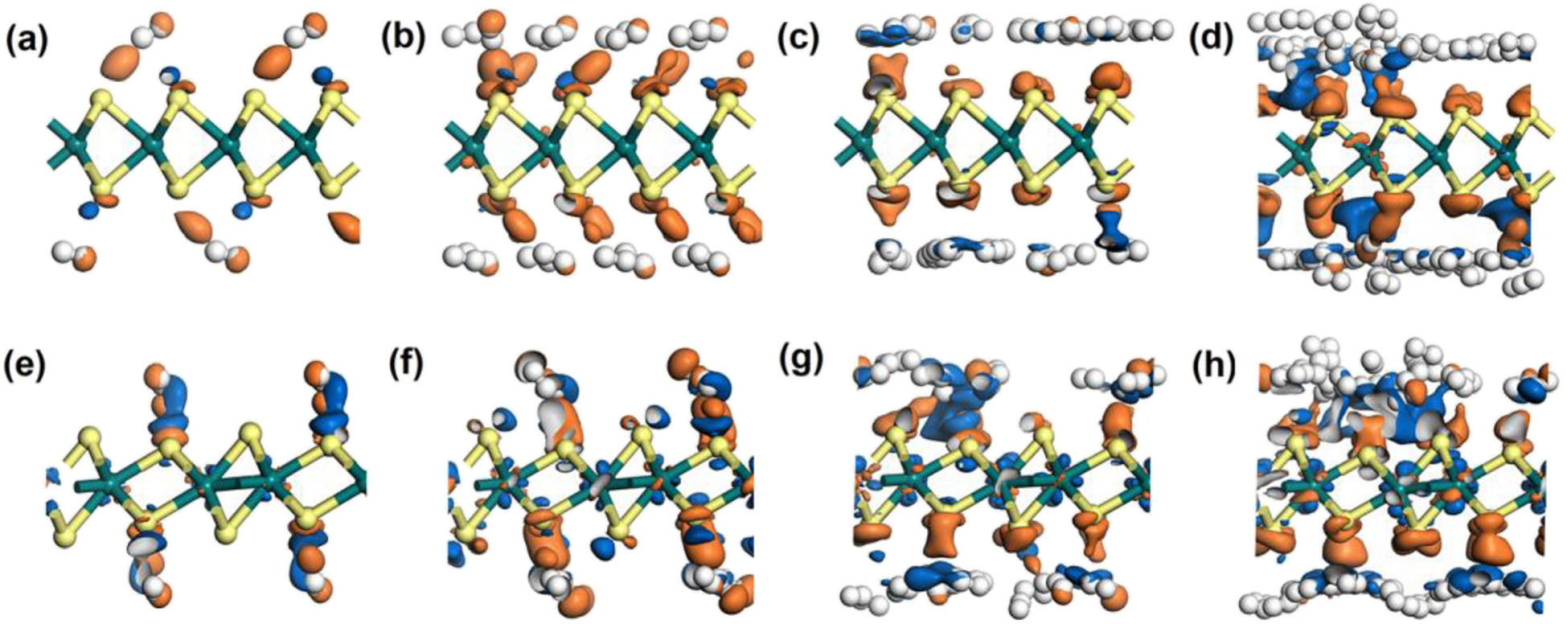
Electronic difference density of **a** 4H_2_ on 2H-WS_2_, **b** 16H_2_ on 2H-WS_2_, **c** 32H_2_ on 2H-WS_2_, **d** 64H_2_ on 2H-WS_2_, **e** 4H_2_ on 1T′-WS_2_, **f** 16H_2_ on 1T′-WS_2_, **g** 32H_2_ on 1T′-WS_2_, and **h** 64H_2_ on 1T′-WS_2_. The isosurface value is taken as 0.002 e/Å

To further explore the interaction between hydrogen molecules and the materials, the electron density difference (EDD) study was also carried out. As shown in Fig. 5 (planforms in Figure S5), the EDD results when 4, 16, 32, and 64 hydrogen molecules adsorbed on 2H or 1T′-WS_2_ were presented. The orange areas represent positive value areas, indicating a tendency to obtain electrons. While the blue areas mean negative areas, representing a depletion of electrons. For both 2H and 1T′-WS_2_, the orange areas were more likely to appear near S atoms, while the blue areas were close to H atoms. The tendency becomes more distinct when 32 or 64 hydrogens were adsorbed, as Fig. 5c, d, g, and h shows. It could also be observed that there were orange and blue areas among the hydrogen molecules when more hydrogen molecules were adsorbed, indicating interaction among the adsorbed H_2_ molecules exists. That adds to the adsorbing force for each hydrogen molecule on the material, increasing the average adsorption energy. Besides, there is another thing that could not be ignored that evident blue areas could be seen when more hydrogen molecules adsorbed on 1T′-WS_2_ (Fig. 5 g, h). While in 2H cases, such a phenomenon is not evident. That manifests that the W atoms also went through a process of electron redistribution. And the W atoms in 1T′-WS_2_ tended to offer more electrons to share the electron supply that mainly provided by the hydrogen molecules than that in 2H-WS_2_ cases. Based on this, the acting force on each hydrogen molecule was weakened to some extent. That could be the reason why the 1T′-WS_2_ could accommodate more hydrogen molecules than the 2H-WS_2_ under the guarantee of the average adsorbing force to be moderate.

## Conclusion

In this paper, hydrogen adsorption models of 2H, 1T, and 1T′ monolayer WS_2_ were constructed. Their adsorption capacity to hydrogen is explored by local density approximation (LDA). Then, by comparing the adsorption energy when multiple hydrogen molecules were adsorbed, it was found that 1T′-WS_2_ could contain more hydrogen molecules than 2H-WS_2_ while the average adsorption energy is in the moderate range (− 0.2 to − 0.6 eV). It can reach the reasonable hydrogen adsorption ratio up to 2.7 wt%, more than that of 2H-WS_2_, which is 2.4 wt%, indicating the influence of phase is apparent for hydrogen storage, and 1T′ phase WS_2_ owns larger hydrogen capacity than the 2H counterpart. Considering all the results calculated in this study, 1T′ phase WS_2_ is a suitable material for hydrogen adsorption applications. It could provide a theoretical reference for studies on highly integrated hydrogen storage materials.

## Supplementary information

**Additional file 1.** Supplementary tables and figures.

## Data Availability

All data are fully available without restriction.
