# Supplementary material for: A WS2 Case Theoretical Study: Hydrogen Storage Performance Improved by Phase Altering
Source: Nanoscale Res Lett. 2020 May 7;15:102. doi: 10.1186/s11671-020-03337-6 (PMC7205947; doi:10.1186/s11671-020-03337-6)
Supplement: Supplementary file 1 — Additional file 1. Supplementary tables and figures. [file 11671_2020_3337_MOESM1_ESM.doc]

**A WS_2_ Case Theoretical Study: Hydrogen Storage Performance Improved by Phase Altering**

Jing Zhou ^a^, Jiamu Cao ^a, b, *^, Jianing Shi ^a^, Yufeng Zhang ^a, b, *^, Junyu Chen ^a^, Weiqi Wang ^a^, Xiaowei Liu ^a, b^

*^a^MEMS Center, Harbin Institute of Technology, 150001, China.*

*^b^Key Laboratory of Micro-systems and Micro-Structures Manufacturing, Ministry of Education, 150001, China.*

**Corresponding authors:*

*caojiamu@hit.edu.cn (Jiamu Cao);*

[*yufeng_zhang@hit.edu.cn*](mailto:yufeng_zhang@hit.edu.cn) *(Yufeng Zhang)*

**Table S1**

Typical preparation methods and stabilization ways of 1T/1T’ WS_2_ in previous studies.

| **Number** | **Type(1T/1T’)** | **Methods** | | **reference** |
| --- | --- | --- | --- | --- |
|  |  | *Preparation* | *Stabilization* |  |
| 1 | 1T | Substitutional rhenium doping in multiwalled WS_2_ nanotubes | Carry the 2H-1T phase transformation in nanotube states | [1] |
| 2 | 1T | Innovative colloidal synthesis method | / | [2] |
| 3 | 1T | Exfoliation of natural WS_2_ by interdigitate tert-butyl-lithium | / | [3] |
| 4 | 1T | Intercalate ammonia ions to 2H WS_2_ | By forming N-WS_2_ nanoribbons | [4] |
| 5 | 1T | Combining the ball milling and chemical Li-intercalation | / | [5] |
| 6 | 1T’ | one-pot colloidal synthesis approach | By positively charge oleylamine surfactant | [6] |
| 7 | 1T’ | one-step solvothermal reaction | By intercalation of amine molecules | [7] |

**Table S2**

The values of total adsorption energy E_ad_ and Hirshfeld charge partitioning results of H atoms for single hydrogen molecule adsorption. 1T’-WS_2_ (v or h means hydrogen molecule on this site is vertical or horizontal).

| Structures | E_ad_(eV) | Hirshfeld charge for H atoms |
| --- | --- | --- |
| 2H-WS_2_ 1 | -0.2213294 | -0.0497 |
| 2H-WS_2_ 2 | -0.2846258 | -0.0593 |
| 2H-WS_2_ 3 | -0.2921198 | -0.0598 |
| 2H-WS_2_ 4 | -0.2706310 | -0.0542 |
| 1T-WS_2_ 1 | -4.9281478 | -0.0563 |
| 1T-WS_2_ 2 | -4.8696760 | -0.0454 |
| 1T-WS_2_ 3 | -5.0405717 | -0.0487 |
| 1T-WS_2_ 4 | -4.9467740 | -0.0571 |
| 1T-WS_2_ 5 | -4.7771327 | -0.0534 |
| 1T'-WS_2_ 1v | -0.3142781 | -0.0615 |
| 1T'-WS_2_ 1h | -0.3111678 | -0.0650 |
| 1T'-WS_2_ 2v | -0.2979186 | -0.0610 |
| 1T'-WS_2_ 2h | -0.3004057 | -0.0618 |
| 1T'-WS_2_ 3v | -0.3069555 | -0.0605 |
| 1T'-WS_2_ 3h | -0.3065555 | -0.0593 |
| 1T'-WS_2_ 4v | -0.2502197 | -0.0588 |
| 1T'-WS_2_ 4h | -0.2765277 | -0.0604 |
| 1T'-WS_2_ 5v | -0.2777468 | -0.0594 |
| 1T'-WS_2_ 5h | -0.2761522 | -0.0597 |
| 1T'-WS_2_ 6v | -0.1967221 | -0.0444 |
| 1T'-WS_2_ 6h | -0.2987621 | -0.0603 |

**Table S3**

The primitive values of E_tot_, E_mat_, and E_hyd_ for single hydrogen molecule adsorption.

| Structures | Etot(Ha) | Emat(Ha) | Ehyd(Ha) |
| --- | --- | --- | --- |
| 2H-WS_2_ 1 | -14890.3582585 | -14889.2142063 | -1.1359179 |
| 2H-WS_2_ 2 | -14890.3605846 |
| 2H-WS_2_ 3 | -14890.3608600 |
| 2H-WS_2_ 4 | -14890.3600703 |
| 1T-WS_2_ 1 | -14889.9339641 | -14888.6169396 |
| 1T-WS_2_ 2 | -14889.9318153 |
| 1T-WS_2_ 3 | -14889.9380956 |
| 1T-WS_2_ 4 | -14889.9346486 |
| 1T-WS_2_ 5 | -14889.9284144 |
| 1T'-WS_2_ 1v | -14889.9921339 | -14888.8446659 |
| 1T'-WS_2_ 1h | -14889.9920196 |
| 1T'-WS_2_ 2v | -14889.9915327 |
| 1T'-WS_2_ 2h | -14889.9916241 |
| 1T'-WS_2_ 3v | -14889.9918648 |
| 1T'-WS_2_ 3h | -14889.9918501 |
| 1T'-WS_2_ 4v | -14889.9897798 |
| 1T'-WS_2_ 4h | -14889.9907466 |
| 1T'-WS_2_ 5v | -14889.9907914 |
| 1T'-WS_2_ 5h | -14889.9907328 |
| 1T'-WS_2_ 6v | -14889.9878138 |
| 1T'-WS_2_ 6h | -14889.9915637 |

**Table S4**

Primitive values of E_tot_, E_ad_, and E_ave_ calculated for different cases when 2 or 3 hydrogen molecules adsorbed on the 1T’ WS_2_.

| Number and arrangement of H_2_ Molecules | | E_tot_ for 1T’-phase WS_2_ (Ha) | E_ad_ (eV) | E_ave_ (eV) |
| --- | --- | --- | --- | --- |
| 2 | 2H_2_-1 | -14891.1428976 | -0.71827 | -0.35913 |
|  | 2H_2_-2 | -14891.1395634 | -0.62754 | -0.31377 |
|  | 2H_2_-3 | -14891.1394409 | -0.62420 | -0.31210 |
| 3 | 3H_2_-1 | -14892.2930246 | -1.10492 | -0.36831 |
|  | 3H_2_-2 | -14892.2903979 | -1.03344 | -0.34448 |
|  | 3H_2_-3 | -14892.2872093 | -0.94668 | -0.31556 |
|  | 3H_2_-4 | -14892.2903295 | -1.03158 | -0.34386 |
|  | 3H_2_-5 | -14892.2868588 | -0.93714 | -0.31238 |

**Table S5**

The primitive values of Etot , Ead, and Eave calculated for multiple hydrogen molecules adsorption for 2H-phase WS2.

| Number of H_2_ Molecules | E_tot_ for 2H-phase WS_2_ (Ha) | E_ad_ (eV) | E_ave_ (eV) |
| --- | --- | --- | --- |
| 2 | -14891.5095281 | -0.6390525 | -0.3195275 |
| 3 | -14892.6602699 | -1.0424152 | -0.3474733 |
| 4 | -14893.8007887 | -1.167600 | -0.29190 |
| 5 | -14894.9475548 | -1.462780 | -0.29256 |
| 6 | -14896.0941991 | -1.754640 | -0.29244 |
| 7 | -14897.2409774 | -2.050150 | -0.29288 |
| 8 | -14898.3876370 | -2.342430 | -0.29280 |
| 12 | -14903.0061937 | -4.380090 | -0.36501 |
| 16 | -14907.5971078 | -5.665560 | -0.35410 |
| 24 | -14916.8563725 | -10.343650 | -0.43099 |
| 32 | -14926.1750845 | -16.639400 | -0.51998 |
| 40 | -14935.4840112 | -22.668860 | -0.56672 |
| 48 | -14944.7828357 | -28.423430 | -0.59215 |
| 49 | -14946.0201507 | -31.182575 | -0.63638 |
| 50 | -14947.1867649 | -32.017848 | -0.64036 |
| 56 | -14954.2247241 | -38.070974 | -0.67984 |
| 64 | -14963.4818631 | -42.691230 | -0.66705 |
|  |  |  |  |

**Table S6**

The primitive values of E_tot_ , E_ad_, and E_ave_ calculated for multiple hydrogen molecule adsorptions for 1T**’**-phase WS_2_.

| Number of H_2_ Molecules | E_tot_ for 1T’-phase WS_2_ (Ha) | E_ad_(eV) | E_ave_(eV) |
| --- | --- | --- | --- |
| 2 | -14891.1407337 | -0.65936 | -0.32690 |
| 3 | -14892.2901698 | -1.0271833 | -0.3423933 |
| 4 | -14893.4382943 | -1.3593325 | -0.3398325 |
| 5 | -14894.5821352 | -1.57491 | -0.314980 |
| 6 | -14895.7296152 | -1.88951 | -0.314920 |
| 8 | -14898.0245923 | -2.51918 | -0.314900 |
| 12 | -14902.6383334 | -4.42581 | -0.368820 |
| 16 | -14907.2407152 | -6.02333 | -0.376460 |
| 20 | -14911.8634480 | -8.17463 | -0.408730 |
| 24 | -14916.4915310 | -10.47152 | -0.436310 |
| 32 | -14925.8234090 | -17.12552 | -0.535170 |
| 40 | -14935.0465514 | -20.82068 | -0.520520 |
| 48 | -14944.3594010 | -26.95689 | -0.561600 |
| 56 | -14953.7033508 | -33.93939 | -0.606060 |
| 64 | -14963.0579679 | -41.21216 | -0.643940 |


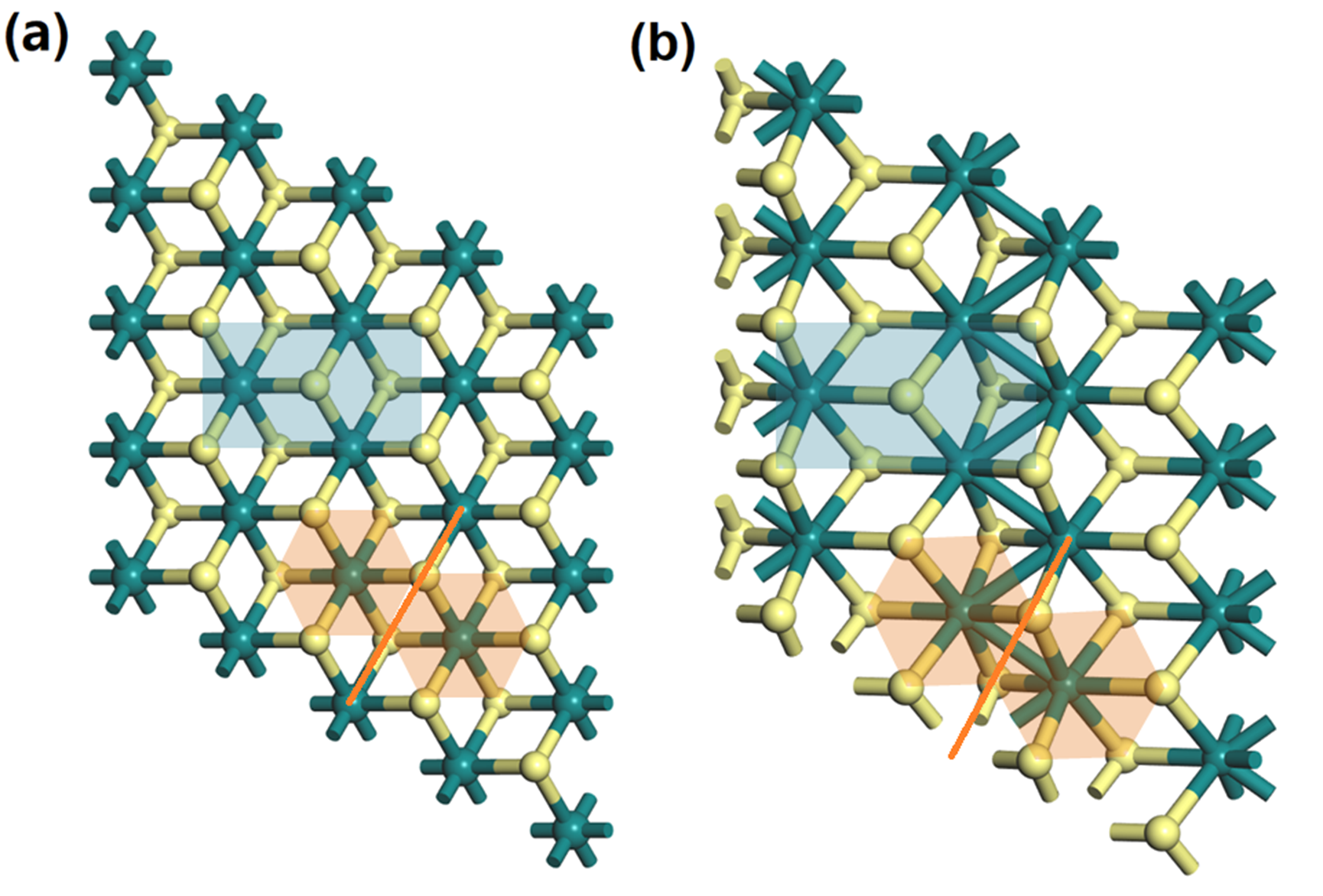


**Figure S1.** Structure units that have symmetry in the (a)1T and (b) 1T’ model.


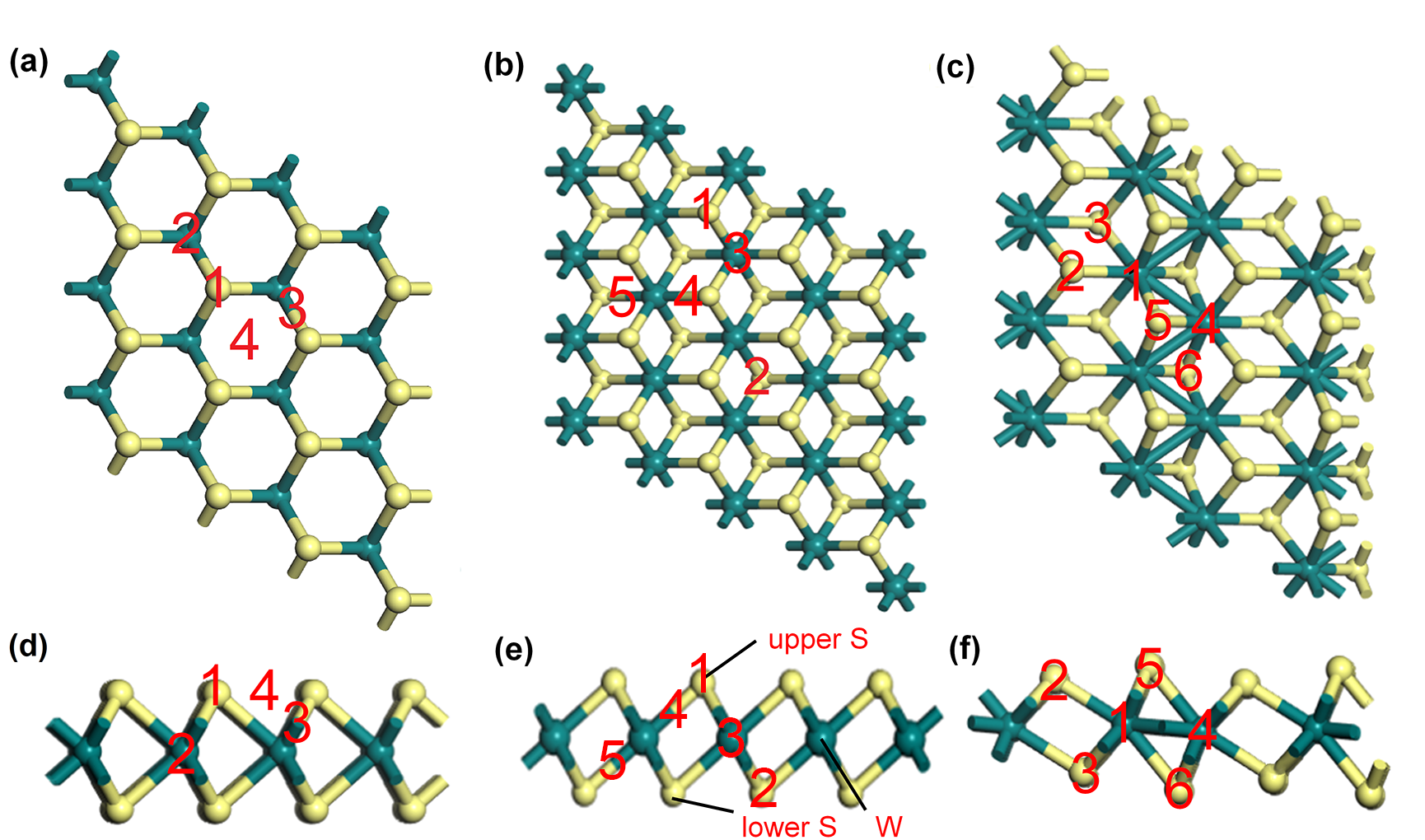


**Figure S2.** The chosen hydrogen placement sites for (a) and (d) 2H-WS_2_ (b) and (e) 1T-WS_2_ (c) and (f) 1T’ WS_2_.


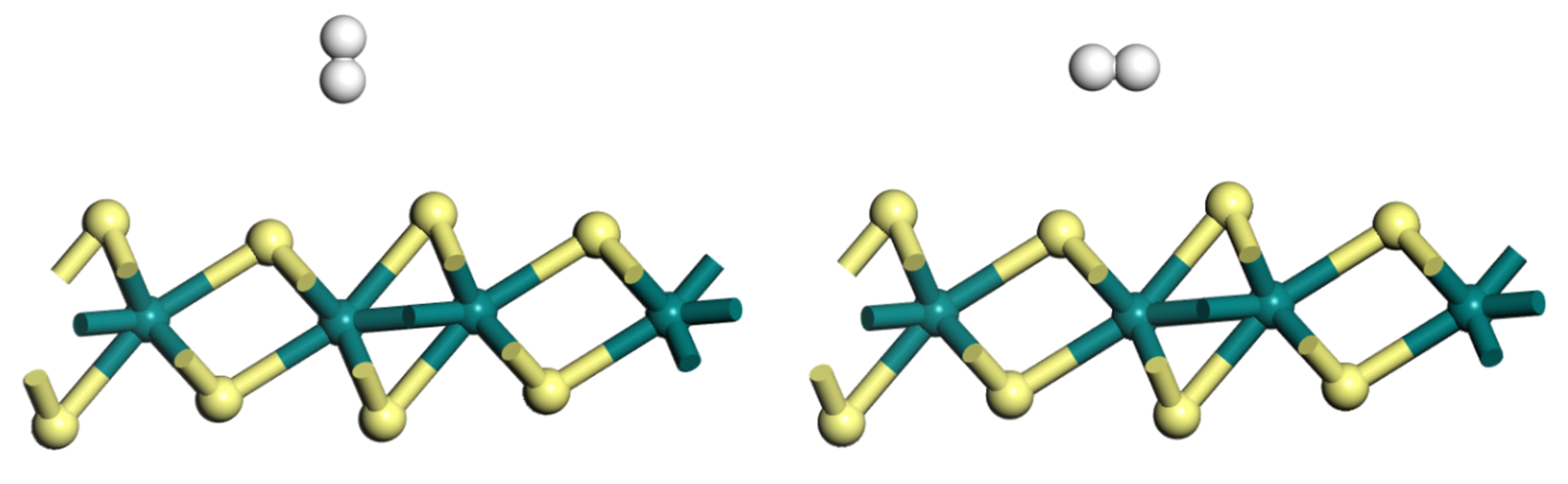


**Figure S3.** Adsorption forms of hydrogen molecules (left)vertically to the materials plane (right) horizontally to the materials plane.


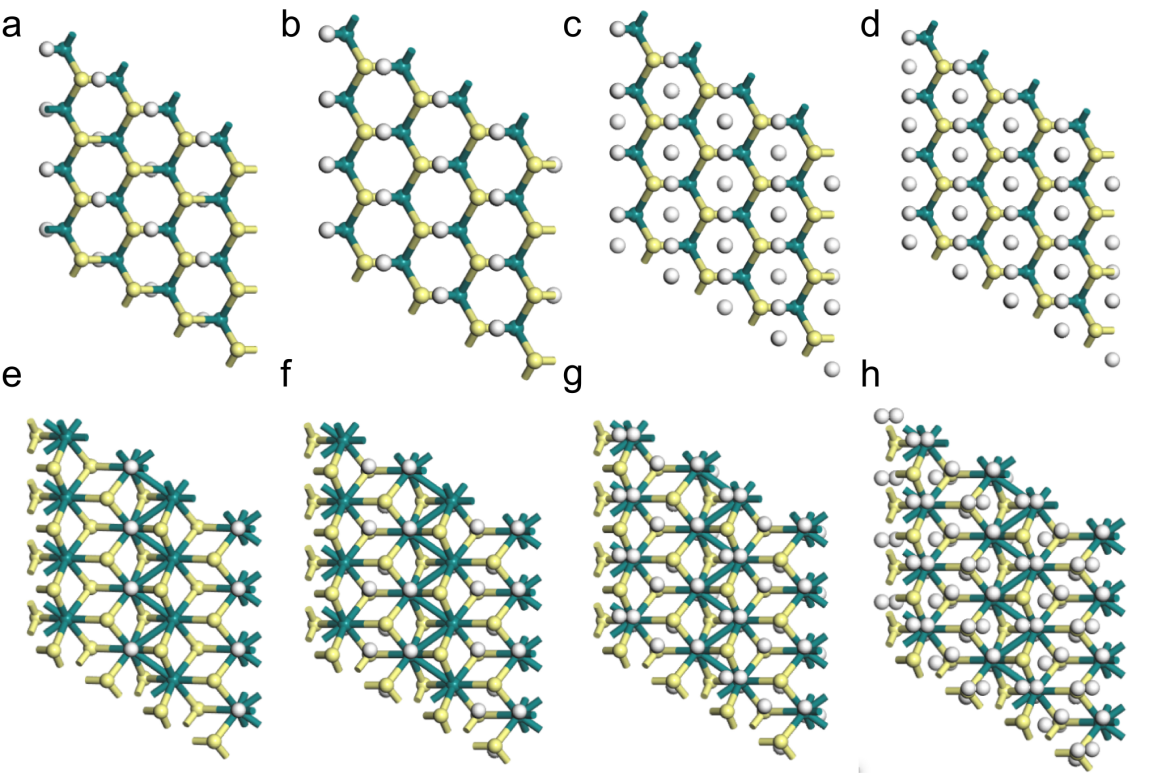


**Figure S4.** Multiple H_2_ adsorbed on 2H-phase WS_2_ and 1T’-phase WS_2_:(a) 16 H_2_ on 2H-phase WS_2_ (b) 32 H_2_ on 2H-phase WS_2_ (c) 48 H_2_ on 2H-phase WS_2_ (d) 64 H_2_ on 2H-phase WS_2_ (e) 16 H_2_ on 1T’-phase WS_2_ (f) 32 H_2_ on 1T’-phase WS_2_ (g) 48 H_2_ on 1T’-phase WS_2_ (h) 64 H_2_ on 1T’-phase WS_2_.


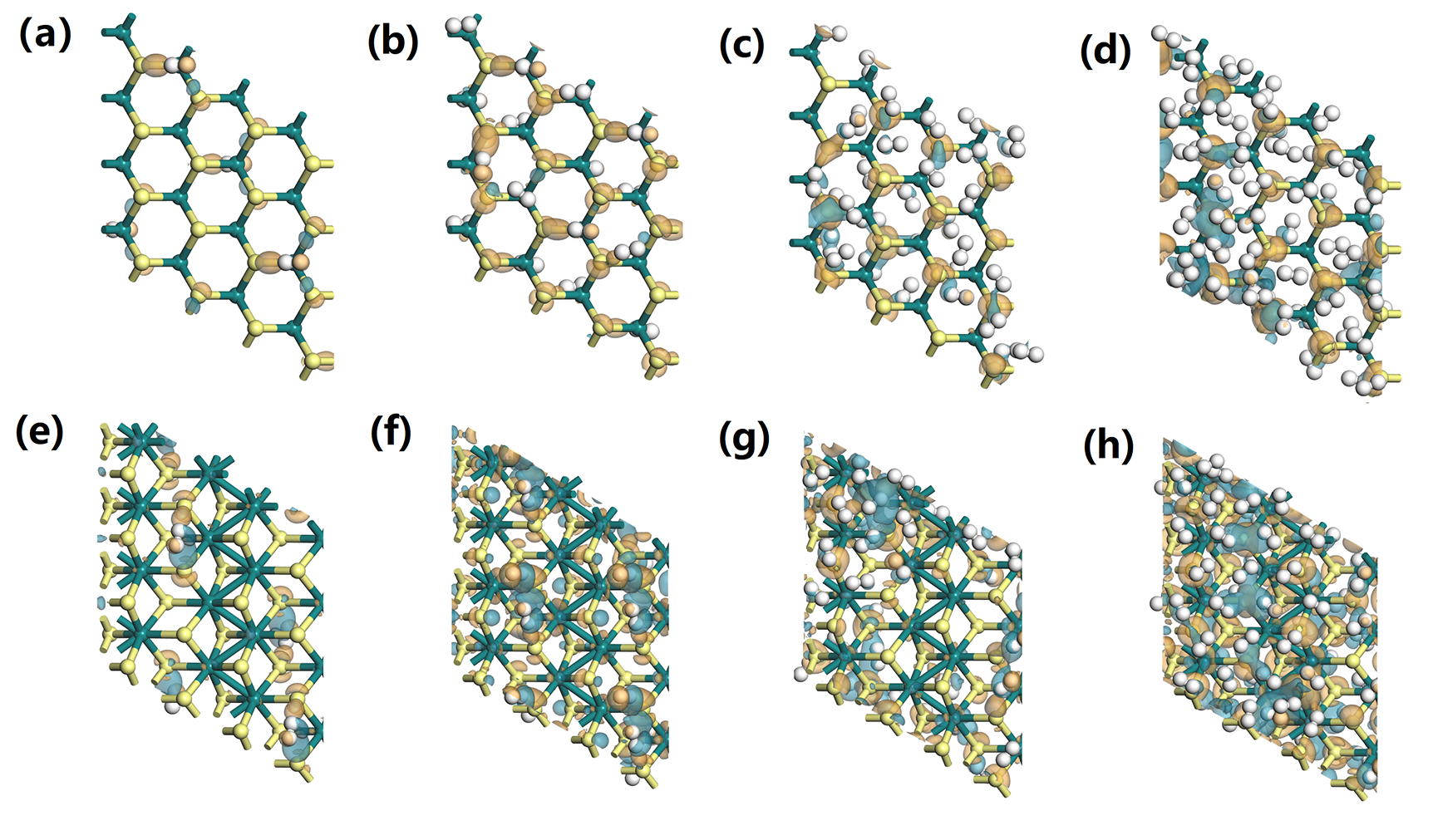


**Figure S5.** Planforms of the Electronic difference density results of (a) 4H_2_ on 2H WS_2_ (b) 16H_2_ on 2H WS_2_ (c) 32H_2_ on 2H WS_2_ (d) 64H_2_ on 2H WS_2_ (e) 4H_2_ on 1T’ WS_2_ (f) 16H_2_ on 1T’ WS_2_ (g) 32H_2_ on 1T’ WS_2_ (h) 64H_2_ on 1T’ WS_2_. The isosurface value is taken as 0.002 e/Å.


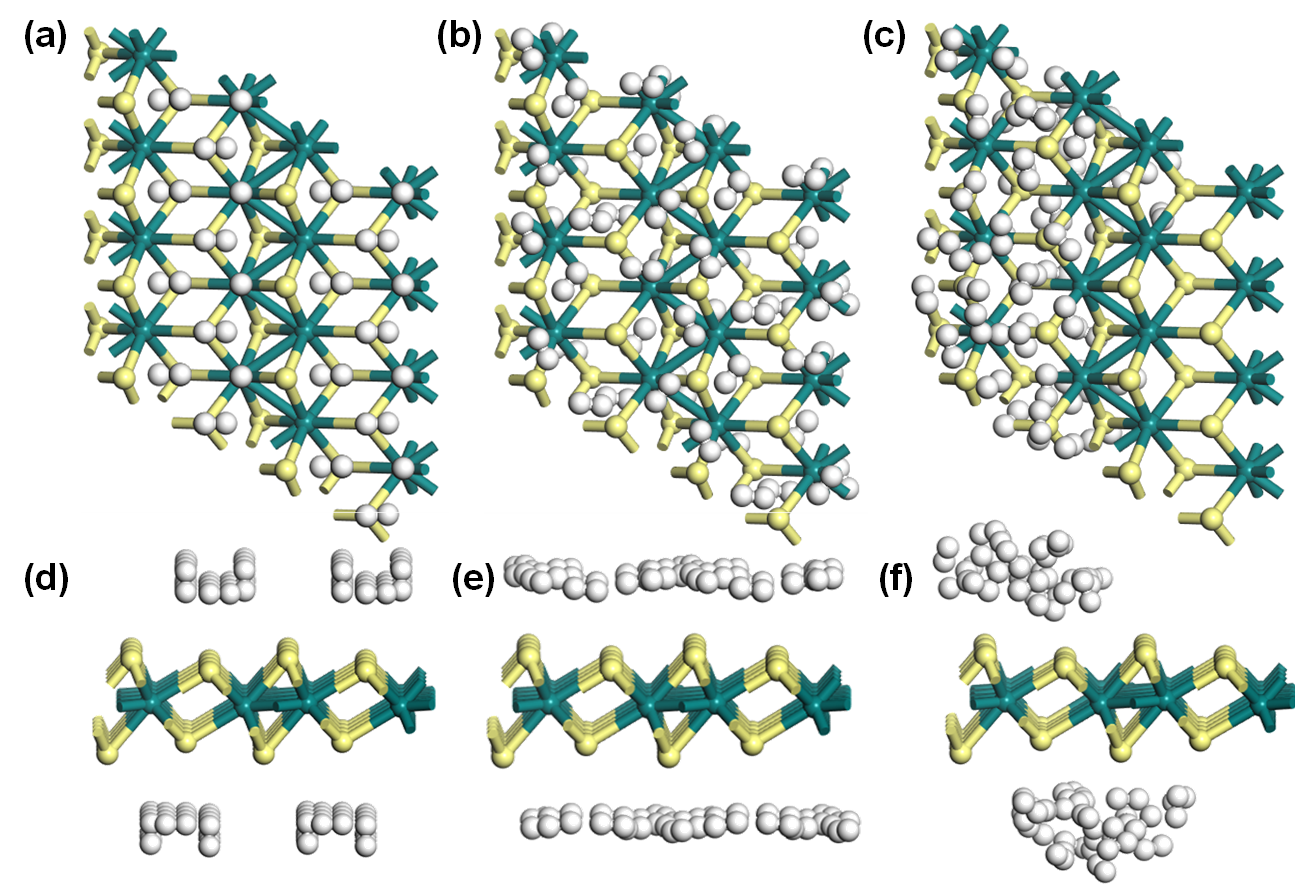


**Figure S6.** Adsorption sites in the case of multiple molecules.

When the number of hydrogen molecules exceeds 32, because the initial height of hydrogen molecules is the same, the hydrogen molecules are relatively dense. If the hydrogen molecular spacing is not considered, only the hydrogen molecule can be placed according to the adsorption energy of single hydrogen, and the localization of hydrogen molecule distribution is easy to occur in the structural optimization. In this way, hydrogen molecular groups with a large number of hydrogen molecules will be formed, and their interaction mode with materials is different from that of one or several hydrogen molecules, which also increases uncertainty for the change of average adsorption energy. To avoid this situation, when the number of hydrogen molecules placed on the surface of 1T'WS_2_ is greater than 32, the distance between the hydrogen molecules and the placed hydrogen molecules will be given priority.

When the number of a gas molecule is more than 16, the stability of the whole system has also been explored by molecular dynamics simulations at 300K using the simple Nose-Hoover heat bath scheme. The time step is set as 2.0 fs for the total 1.0 ps simulation time. In Figure S7(a), the total energy is between -14907.1290 and -14907.1268eV. In Figure S7(b), the total energy is between -14925.6628 and -14907.6600eV In Figure S7(c), the total energy is between -14944.1575 and -14907.1520eV Figure S7(d), the total energy is between -14962.8110 and -14907.8060eV. Therefore, after 500 steps of molecule dynamics simulation, there is no geometry buckling emerging, and the overall energy also remains almost constant. Hence, the whole system has excellent stability when the hydrogen concentration is high.


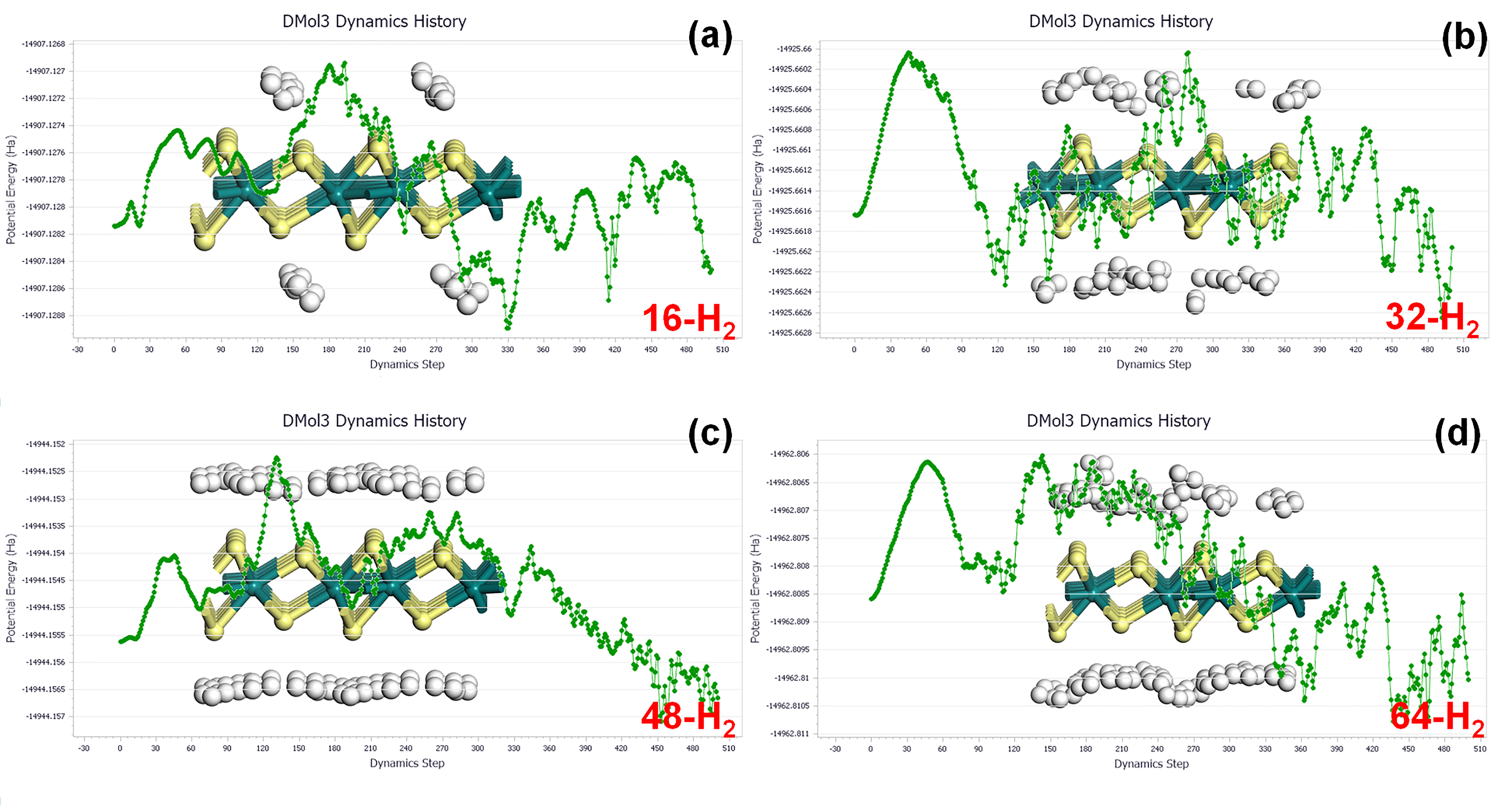


**Figure S7.** Molecule dynaics simaulations at 300K (a) 16 H_2_ (b) 32 H_2_ (c) 48 H_2_ (d) 64 H_2_.

**Reference**

[1] Andrey N. Enyashin, Lena Yadgarov, Lothar Houben, Igor Popov, Marc Weidenbach, Reshef Tenne, Maya Bar-Sadan and Gotthard Seifert, New Route for Stabilization of 1T-WS_2_ and MoS_2_ Phases, Journal of Physical Chemistry C,115(2011) 24586-24591.

[2] Benoit Mahler, Veronika Hoepfner, Kristine Liao, and Geo rey A. Ozin, Colloidal Synthesis of 1T-WS_2_ and 2H-WS_2_ Nanosheets: Applications for Photocatalytic Hydrogen Evolution, Journal of the American Chemical Society, 136(2014) 14121-14127.

[3] Carmen C. Mayorga-Martinez, Adriano Ambrosi, Alex Yong Sheng Eng, Zdenek Sofer, and Martin Pumera, Metallic 1T-WS_2_ for Selective Impedimetric Vapor SensingAdvanced Functional Materials, 25(2015) 5611-5616.

[4] Qin Liu, Xiuling Li, Zhangru Xiao, Yu Zhou, Haipin Chen, Adnan Khalil, Ting Xiang, Junqing Xu, Wangsheng Chu, Xiaojun Wu, Jinlong Yang, Chengming Wang, Yujie Xiong, Chuanhong Jin, Pulickel M. Ajayan, and Li Song, Stable Metallic 1T-WS_2_ Nanoribbons Intercalated with Ammonia Ions: The Correlation between Structure and Electrical/Optical Properties, Advanced Materials, 27(2015) 4837-4844.

[5] Chaoliang Tan, Zhimin Luo, Apoorva Chaturvedi, Yongqing Cai, Yonghua Du, Yue Gong, Ying Huang, Zhuangchai Lai, Xiao Zhang, Lirong Zheng, Xiaoying Qi, Min Hao Goh, Jie Wang, Shikui Han, Xue-Jun Wu, Lin Gu, Christian Kloc, and Hua Zhang, Preparation of High-Percentage 1T-Phase Transition Metal Dichalcogenide Nanodots for Electrochemical Hydrogen Evolution, Advanced Materials, 30(2018) 1705509.

[6] Zhengqing Liu, Na Li, Cong Su, Hongyang Zhao, Lingling Xu, Zongyou Yin, Ju Li,Yaping Du, Colloidal synthesis of 1T' phase dominated WS_2_ towards endurable electrocatalysis, Nano Energy, 50(2018) 176-181.

[7] In Hye Kwak, Ha z Ghulam Abbas, Ik Seon Kwon, Yun Chang Park, Jaemin Seo, Min Kyung Cho, Jae-Pyoung Ahn, Hee Won Seo, Jeunghee Park and Hong Seok Kang, Intercalation of cobaltocene into WS_2_ nanosheets for enhanced catalytic hydrogen evolution reaction, Journal of Materials Chemistry A, 7(2019) 8101.
